# Overcoming stress limitations in SiN nonlinear photonics via a bilayer waveguide

**DOI:** 10.1515/nanoph-2024-0457

**Published:** 2025-02-14

**Authors:** Karl J. McNulty, Shriddha Chaitanya, Swarnava Sanyal, Andres Gil-Molina, Mateus Corato-Zanarella, Yoshitomo Okawachi, Alexander L. Gaeta, Michal Lipson

**Affiliations:** Department of Electrical Engineering, Columbia University, New York, NY 10027, USA; Department of Applied Physics & Applied Mathematics, Columbia University, New York, NY 10027, USA; Department of Electrical Engineering and Department of Applied Physics & Applied Mathematics, Columbia University, New York, NY 10027, USA

**Keywords:** silicon nitride, film stress, nonlinear, frequency comb

## Abstract

Silicon nitride (SiN) formed via low pressure chemical vapor deposition (LPCVD) is an ideal material platform for on-chip nonlinear photonics owing to its low propagation loss and competitive nonlinear index. Despite this, LPCVD SiN is restricted in its scalability due to the film stress when high thicknesses, required for nonlinear dispersion engineering, are deposited. This stress in turn leads to film cracking and makes integrating such films in silicon foundries challenging. To overcome this limitation, we propose a bilayer waveguide scheme comprised of a thin LPCVD SiN layer underneath a low-stress and low-index PECVD SiN layer. We show group velocity dispersion tuning at 1,550 nm without concern for film-cracking while enabling low loss resonators with intrinsic quality factors above 1 million. Finally, we demonstrate a locked, normal dispersion Kerr frequency comb with our bilayer waveguide resonators spanning 120 nm in the c-band with an on-chip pump power of 350 mW.

## 1 Introduction

**Figure 1:**
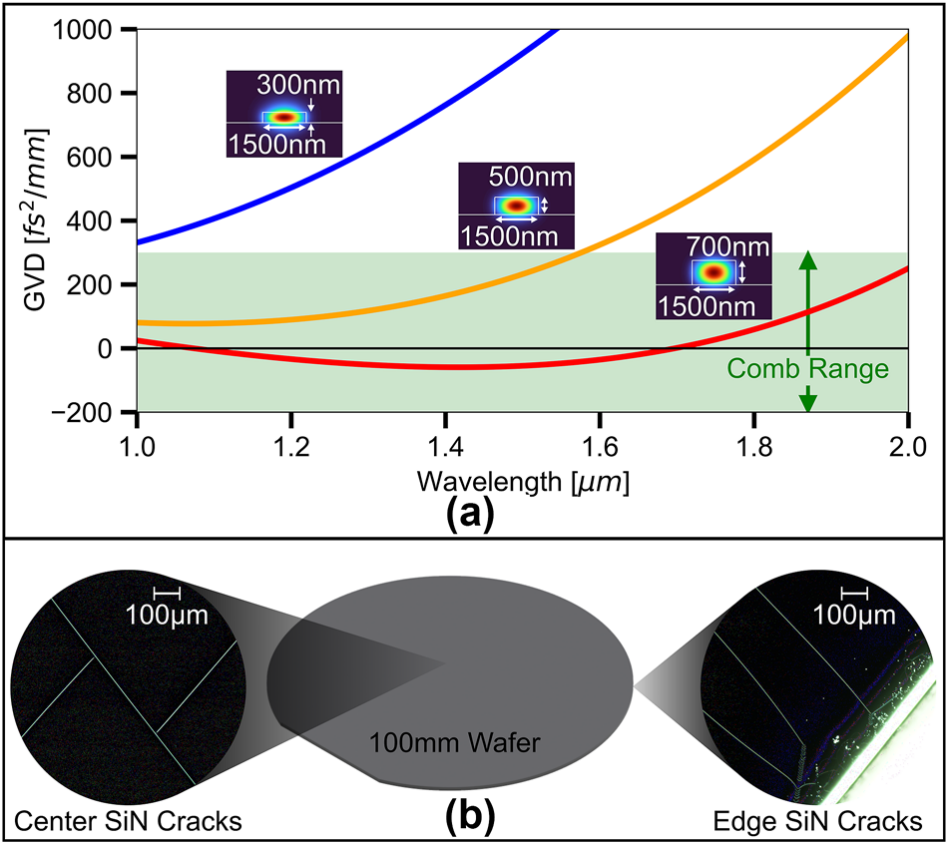
Thickness dependent waveguide dispersion and film cracking in SiN. (a) Dispersion comparison of a 1.5 μm wide LPCVD SiN waveguide with various thicknesses. The green shaded area indicates a general GVD range which has been shown to generate microcombs. (b) Visible film cracking seen under a dark field microscope of a 730 nm thick LPCVD SiN film on a 100 mm wafer. Cracks propagate from the edge of a wafer (right) and pass through the center of the wafer (left).

Low pressure chemical vapor deposition (LPCVD) silicon nitride (SiN) films have emerged as an ideal candidate for optical non-linear applications, owing to SiN’s low optical loss and high *n*_2_ value [1]. Despite the desirable characteristics, LPCVD SiN waveguides often require thick films (>400 nm) to satisfy the group velocity dispersion (GVD) requirements for nonlinear processes in the telecom range. Such thick SiN films are plagued by high tensile stress and subsequent film cracking, thereby lowering the photonic device yield and limiting their applicability in large-scale systems. Figure 1a shows the simulated GVD for an LPCVD SiN waveguide with a 1.5 μm width and various heights. As an example of dispersion needed for nonlinear processes, we highlight the dispersion range that facilitates comb formation in the telecom wavelength range [2], [3], [4], [5], [6], [7], [8], [9], [10], [11]. As Figure 1a indicates, the waveguide GVD in the c-band only reaches the target normal and anomalous GVD range when the waveguide thickness is beyond 500 nm, above the typical thickness limit of 400 nm in foundries [12]. Figure 1b shows an example of the extreme cracking in 700 nm SiN film which forms at the edge of a 100 mm wafer (right) and propagates to the center of the wafer (left). Such cracking is almost unavoidable in LPCVD SiN films above the thickness threshold of 400 nm and is a key limiting factor of wider integration of SiN based nonlinear photonics.

To mitigate the material stress limitation, fabrication approaches consist of stress relief trenches prior to SiN deposition [13], [14], [15], [16], [17], [18] or the use of chemical mechanical planarization (CMP) for a photonic damascene process [19], [20], [21]. Stress relief trenches take up valuable wafer area leading to potential compromises in device density. High film stress at large thicknesses can also cause cracking to occur at the trench locations, making such trenches not always reliable against cracking (see Supplementary material). Although the damascene process has been scaled to 200 mm wafer sizes, it is hindered by variation in height from CMP and variation in width from thermal reflow processing steps. As such, waveguide dimensions and coupling gap sizes may be difficult to control leading to inaccurate waveguide dispersion and coupling [22]. Thermal cycling throughout deposition may also be employed to overcome cracking once LPCVD films surpass 400 nm in thickness. However, this has only recently been scaled to 150 mm wafers [14], and the cycled anneal steps lead to intermediate layers within the LPCVD SiN. Circumventing LPCVD SiN through the use of sputtered SiN [23], [24], [25], PECVD SiN [26], and deuterated PECVD SiN [27], [28], [29], [30] for stress-free, nonlinear devices has also been shown. In particular, deuterated PECVD SiN is a promising new platform for SiN based nonlinear technology as it provides a path towards low-loss resonators with low temperature depositions. The tools and gases required for deuterated PECVD SiN are expensive though, making deuterated PECVD films far less available compared to normal PECVD SiN. Additionally, deuterated SiN resonators, and all other high confinement methods, have not reached the lowest levels of loss achieved via LPCVD SiN processes [22], [31], [32].

Thin LPCVD SiN films below 400 nm, in contrast to thick LPCVD SiN films, do not suffer from stress and are readily available in foundries. Nonlinear processes may be realized with such thin films via mode crossing techniques [33], [34], [35]. However, devices based on thinner nitride films lack high modal confinement and the ability for broadband dispersion engineering [36].

**Figure 2:**
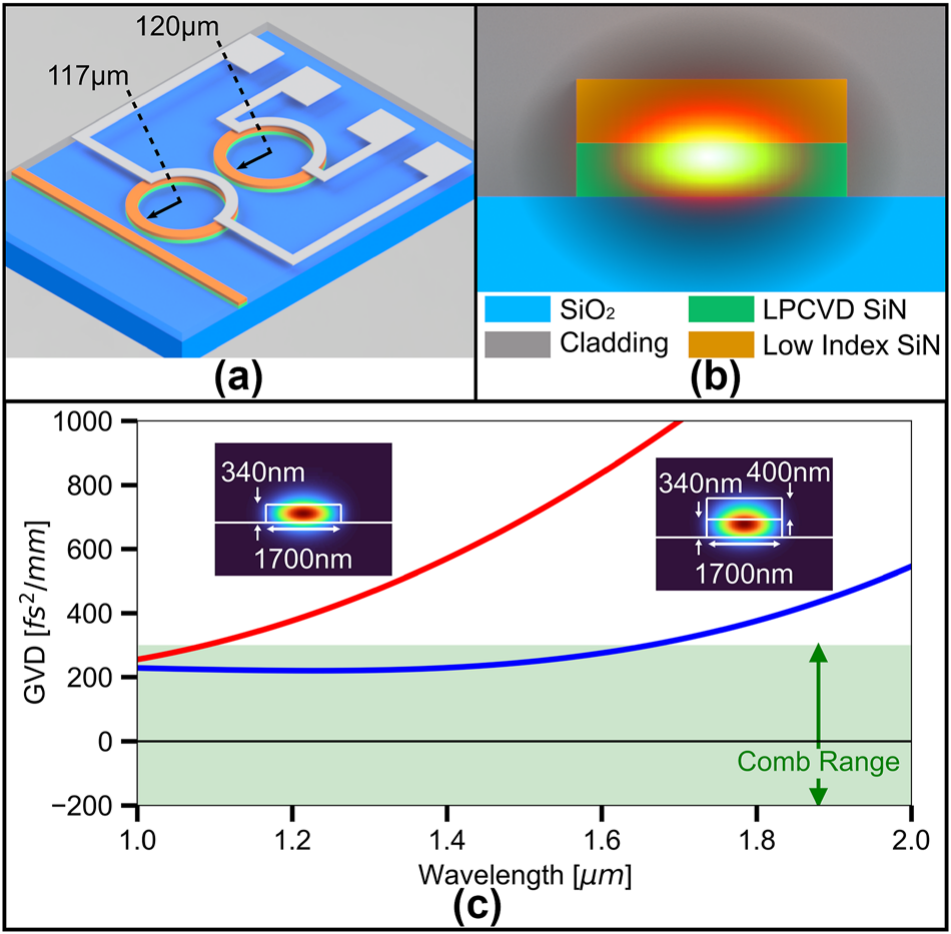
Bilayer waveguide design and dispersion. (a) 3D rendering of the bilayer design consisting of dual ring resonators and on-chip heaters for resonance detuning control. (b) Cross-section of the 1700 nm × 740 nm waveguide along with an overlay of the optical mode. (c) Waveguide GVD comparison of an unaided 1700 nm × 340 nm LPCVD SiN waveguide with that of the bilayer design. The addition of the low index SiN layer brings the dispersion down within the range of demonstrated combs without the tradeoff of high film stress.

Here we endow a foundry compatible, thin film LPCVD SiN with the desired GVD via a stress-free bilayer waveguide configuration. Shown in Figure 2b, we deposit a low index PECVD SiN (index of 1.78 at 1,550 nm) on a thin film of LPCVD SiN. The low index SiN layer enables stress free tuning of the overall dispersion of the composite waveguide, while maintaining low loss for the whole composite waveguide. It also ensures high modal overlap with the bottom LPCVD SiN to utilize its higher nonlinearity as the low index SiN is less dense and, therefore, has a lower relative *n*_2_ [37] (see Supplementary material for film details). As our fabrication is subtractive and the film cracking is addressed via a bilayer film stack, our design offers the simplest approach to fabrication of dispersion engineered SiN waveguides. There is no concern for dishing or waveguide dimension variations from CMP and thermal reflow, nor need for stress-relief trenches which can limit device density and may not completely prevent cracking. Moreover, although we select a top film with a large index contrast to demonstrate the robustness of the approach, our method is adaptable to other variants of SiN films for the top layer including deuterated PECVD SiN. As an example of application, we use our bilayer waveguides to construct dual resonators, depicted in Figure 2a, which are commonly used for comb generation [10]. We choose a waveguide width of 1700 nm for our design, an LPCVD SiN layer of 340 nm in thickness, and a low index SiN layer of 400 nm in thickness. As Figure 2c shows, our design allows significant control over the waveguide GVD compared to the unaided thin LPCVD SiN waveguide of the same width. From dispersion simulations, the bilayer waveguide exhibits a GVD within the c-band sufficiently low to where normal GVD SiN microcombs have been demonstrated [2], [4], [5], [6], [7], [10]. In contrast we see that the 340 nm thick LPCVD SiN waveguide exhibits very high normal GVD and fails to reach the generally required dispersion for microcomb generation.

## 2 Results and discussion

**Figure 3:**
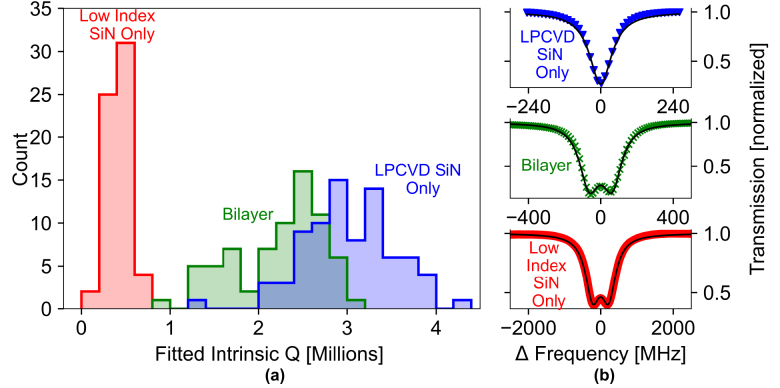
Intrinsic quality factors for resonators of each waveguide type. (a) Histogram of the fitted intrinsic quality factors measured across a spectrum of 1,500 nm–1,600 nm for three separate ring resonator devices (0.2 million bin width). All three rings are 150 μm in radius and have cross-sections of 2000 nm × 730 nm. From left to right; the average intrinsic quality factors are 425 thousand, 2.23 million, and 3.04 million, corresponding to average loss rates of approximately 72.7 dB/m, 14.8 dB/m, and 11.1 dB/m, respectively. (b) Sample resonance fits for each of the different rings.

We experimentally validate that our waveguide design enables low loss resonators comparable to the loss levels of single-core LPCVD SiN resonators. To directly compare device loss, we fabricate identical single ring resonators with (a) standard 730 nm thick LPCVD SiN waveguides, (b) our bilayer waveguides with the thicknesses specified earlier, and (c) 730 nm thick low index SiN only waveguides. To fabricate the 730 nm thick LPCVD waveguides for comparison, we follow the trench and thermal annealing techniques detailed in [16]. We choose the ring radius to be 150 μm and the waveguide width to be 2000 nm for all devices tested. To minimize difference in performance attributed to process variation, these waveguides receive the same process steps and are fabricated in parallel with each other. For loss characterization, we selected one undercoupled ring for each waveguide type and measured roughly 50 resonances across a spectrum of 1,500 nm–1,600 nm. We then employ a coupled mode model with backscattering [38] to fit each individual resonance and extract the intrinsic quality factor as a measurement of cavity loss. Figure 3 shows a histogram of the fitted intrinsic quality factors for each device spectrum. Despite being composed of films of considerably different indices, our bilayer waveguides exhibit only a slight drop in loss compared to LPCVD SiN waveguides and retain intrinsic quality factors well above 1 million across the c-band. This is in stark comparison to the single-core, low index SiN waveguide which has much lower intrinsic quality factors in the range of 100 k - 600 k. Thus, our dispersion tuning design maintains low optical loss while not suffering from film stress.

**Figure 4:**
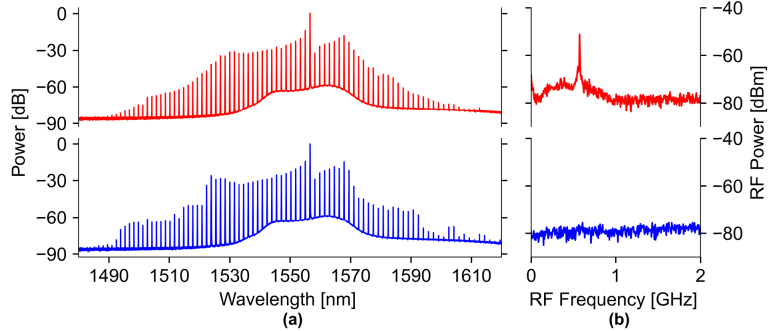
Low noise frequency comb generation in a bilayer waveguide resonator. (a) Optical spectrum analyzer (OSA) spectrum of the unlocked (top) and locked (bottom) comb states of our bilayer waveguide resonator (normalized to the pump line). (b) Associated RF frequency noise measured via an electrical spectrum analyzer (ESA).

To demonstrate the practicality of our bilayer approach for nonlinear photonic applications, we demonstrate a low-noise, Kerr frequency comb spanning approximately 120 nm with 350 mW of on-chip pump power. As our bilayer waveguide has GVD shown in Figure 2b, we employ the dual ring configuration, as mentioned above, to enable comb generation in the normal dispersion regime. For our comb device, we choose a width of 1700 nm for our bilayer waveguide with a main ring of radius 120 μm and an auxiliary ring of radius 117 μm (as shown in Figure 2a). Pumping around 1,557 nm and following [10], we utilize our fully integrated, on-chip microheaters to tune the main ring and auxiliary ring frequency detunings and navigate to a low noise comb state. Figure 4 shows both the unlocked state (top) prior to tuning the microheater currents, and the low-noise, locked state (bottom) of the comb after tuning the microheater currents. Once locked, we observe the characteristic low RF frequency noise associated with such a locked state [39], in contrast to the high RF frequency noise seen in the unlocked state.

## 3 Conclusions

Our approach to dispersion engineering provides a solution to the film stress plaguing SiN technology. By decoupling the waveguide dispersion from the thickness of a single LPCVD SiN layer, we enable dispersion engineered devices with low optical loss and without large film stress during fabrication. We generate coherent, low-noise combs using our new waveguide design and show that our approach is compatible with mode crossing techniques previously used for microcomb generation. Moreover, this approach is adaptable to other materials, as various PECVD Si_*x*_N_*y*_ films or possibly TiO_2_ could be utilized as the dispersion tuning top layer.

## 4 Fabrication methods

We fabricate our devices starting from 100 mm wafers with 4 μm of wet thermal oxide. We then deposit the 340 nm of bottom SiN via LPCVD and follow this by depositing the 400 nm layer of low index SiN via PECVD. The LPCVD SiN film is deposited at 800 °C using a mixture of NH_3_ and DCS (SiH_2_Cl_2_). The PECVD top film is deposited at 350 °C, and we select an appropriate gas mixture of NH_3_, SiH_4_, and N_2_ which yields our low index film. We utilize an oxide hardmask alongside electron beam lithography to define our waveguides and etch them with a plasma dry etch. The waveguides are then annealed in an argon environment for 3 h and subsequently clad with a high temperature silicon dioxide. The platinum heaters are defined on the cladding oxide through a lift off process, and we perform a final deep silicon etch prior to dicing to enable low loss edge couplers. More details of our fabrication can be found in [40].

## Supplementary Material

Supplementary Material Details
