# Supplementary material for: Overcoming stress limitations in SiN nonlinear photonics via a bilayer waveguide
Source: Nanophotonics. 2025 Feb 14;14(23):3921–6. doi: 10.1515/nanoph-2024-0457 (PMC12617702; doi:10.1515/nanoph-2024-0457)
Supplement: Supplementary file 1 — Supplementary Material Details [file j_nanoph-2024-0457_suppl_001.pdf]

Overcoming stress limitations in SiN nonlinear photonics via a bilayer waveguide: supplemental document

1. CRACKING AT TRENCH LOCATIONS

Creating trenches in the oxide prior to LPCVD SiN deposition aids in preventing cracks, but this method can still yield cracks in the film. Figure S1 depicts cracking in a 730 nm thick LPCVD SiN film which forms at a stress relief trench. The trench is made in a similar manner to [1].

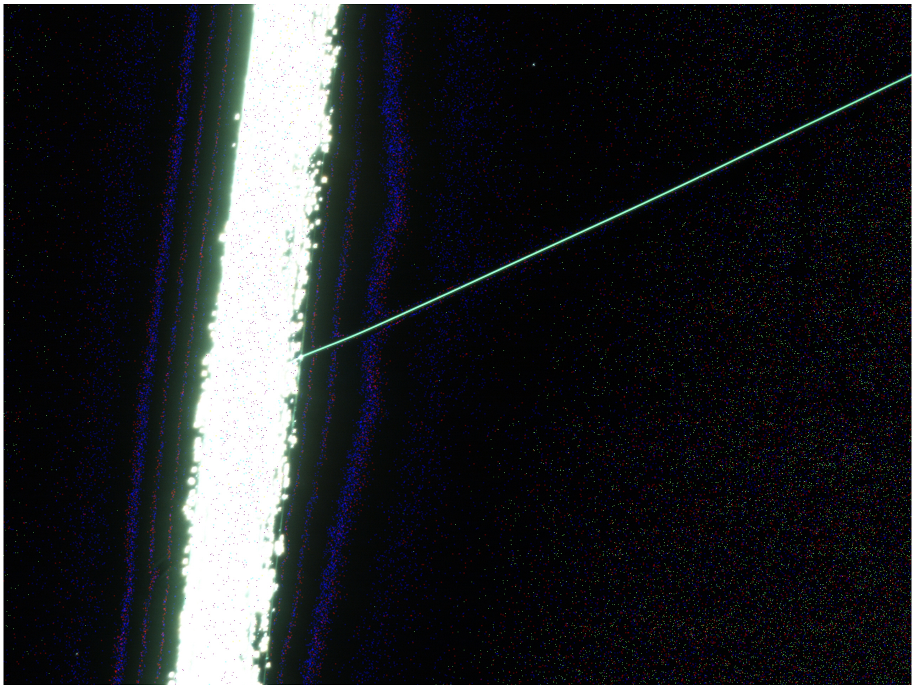

Fig. S1. Dark field microscope image of a crack starting at one of the stress relief trenches. The LPCVD SiN film is 730 nm thick and sits on top of a 4 μm thick oxide layer.

2. EXPERIMENTAL SETUP

Figure S2 shows the schematic of our test setup for the dual ring frequency comb. While the optical spectrum analyzer (OSA) allows us to monitor the frequency comb spectrum while tuning the heaters, the fast photodiode (FPD) and electrical spectrum analyzer (ESA) enables us to determine when the comb is locked. In accordance with [2], the low RF noise spectrum in the ESA alongside a broad OSA optical spectrum is indicative of a locked comb state. For single ring, anomalous comb measurements, the two SMs are replaced with a single arbitrary waveform generator (AWG) to control the single ring heater. Sending the correct waveform from the AWG to the on-chip heater allows us to lock anomalous, single ring combs.

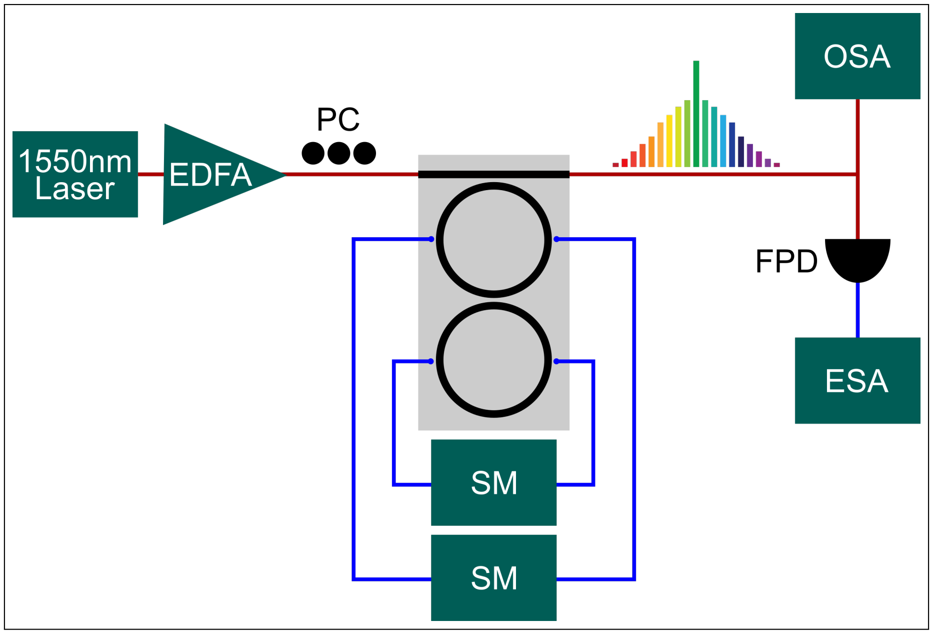

Fig. S2. Experimental setup for testing the dual ring microcomb. A tunable laser around 1550 nm is first sent through an erbium doped fiber amplifier (EDFA) and then a polarization controller (PC) before being coupled to the photonic chip. The frequency comb light is then coupled back to a fiber which is then split and routed to an optical spectrum analyzer (OSA) and fast photodiode (FPD). An electrical spectrum analyzer (ESA) is then connected to the FPD to read out the RF noise spectrum. The microresonator comb is controlled via two source meters (SM) connected to the on-chip heaters.

3. LOW INDEX PECVD SIN FILM CHARACTERIZATION

We provide further analysis on the PECVD SiN film we use for our top dispersion tuning layer within the bilayer design. Figure S3 indicates the index of our PECVD SiN film is consistently 0.2 lower than LPCVD SiN. In order to identify the cause of the lower index, we perform X-ray photoelectron spectroscopy (XPS) measurements to determine atomic composition, as shown in Figure S4. The analysis of the XPS data in Table S1 indicates the two SiN films are very similar in atomic composition, thus we propose the refractive index difference stems from density differences. To test this claim, we use X-ray reflectivity (XRR) to measure film density as seen in Figure S5. The theoretical fit results in Table S2 support our claim and reveal a lower density for the low index PECVD SiN. Due to the porosity of the low index PECVD SiN, we suspect that the film shrinks in width during the high temperature annealing step. Evidently, this does not seem to have a large impact on the design, as our bilayer waveguides still yield low loss resonators capable of generating locked combs.

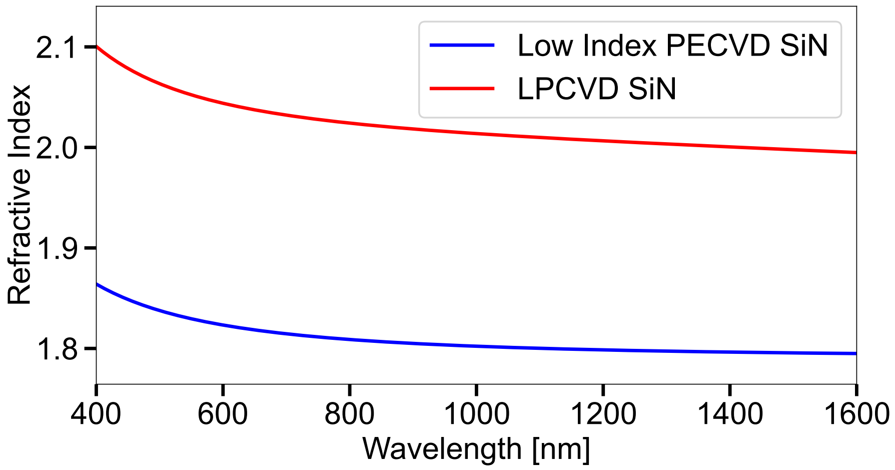

Fig. S3. Refractive index of LPCVD SiN and the low index PECVD SiN. LPCVD SiN index provided by [3]. The low index PECVD SiN curve is obtained via variable angle ellipsometry on a Woollam RC2 Ellipsometer. Ellipsometry data is fitted with a Cauchy profile to extract the index curve.

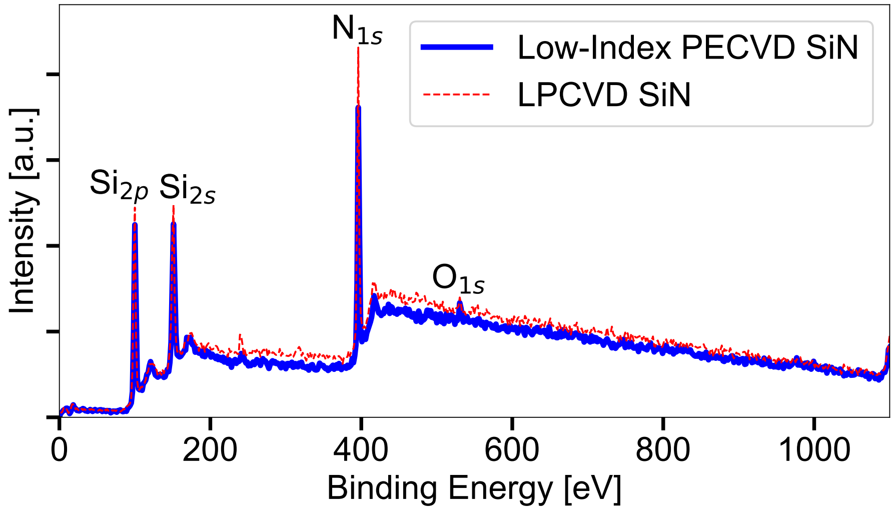

Fig. S4. Raw XPS data collected on LPCVD SiN and the low index PECVD SiN. Data is collected after argon milling roughly 100 nm into the film in order to prevent surface contamination or surface oxidation effects in the data.

Material	Silicon Atomic %	Nitrogen Atomic %	Oxygen Atomic %
LPCVD SiN	46.5	50.7	2.8
Low Index PECVD SiN	47.7	49.2	3.1

Table S1. Extracted XPS elemental composition of LPCVD SiN and the low index PECVD SiN. The composition of both is similar, indicating the index contrast between the two materials does not stem from elemental composition differences.

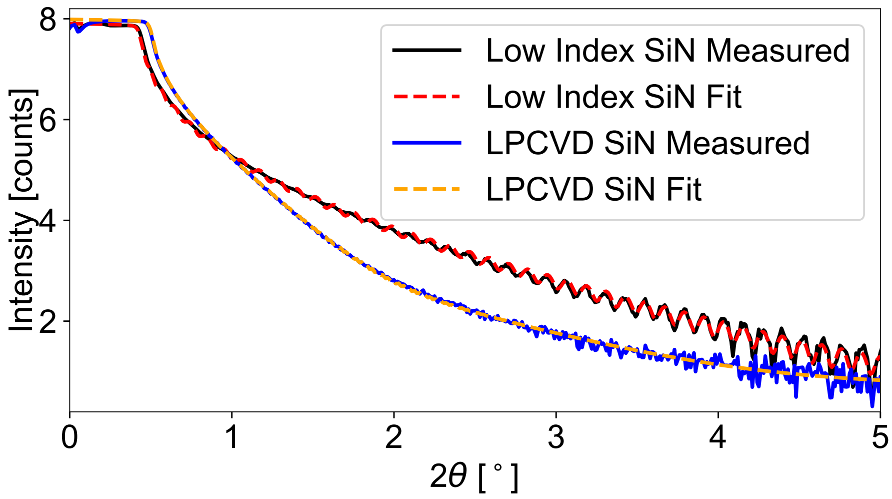

Fig. S5. XRR analysis of an LPCVD SiN film and the low index PECVD SiN film along with the theoretical fits for both. The low index SiN film shows a critical angle (point of initial intensity drop) at a lower 2θ compared to LPCVD SiN, indicating a lower density [4].

Material	Thickness [nm]	Density [g/cm^3]	Surface Roughness [nm]
LPCVD SiN	263	2.91	1.58
Low Index PECVD SiN	73	2.15	0.54

Table S2. Fitted XRR parameters of the LPCVD SiN film and the low index PECVD SiN film. The fit is done in accordance with theory presented in [4]. We attribute the low index of the PECVD SiN to the lower density of the film compared to LPCVD SiN.

4. EXTENSION TO ANOMALOUS DISPERSION

Although our low index PECVD SiN film does not allow us to reach the anomalous dispersion regime, the use of a different and higher index PECVD SiN film would allow for anomalous waveguide GVD. A wide range of index variation is achievable via alteration of PECVD deposition parameters, particularly the gas flows [5]. In Figure S6, we show the simulated GVD of a 1.5 μm wide waveguide with a 340 nm bottom LPCVD SiN layer and a 400 nm top PECVD SiN layer. By changing the refractive index of the top film within the range of realizable PECVD films, we observe that anomalous dispersion can achieved with our design.

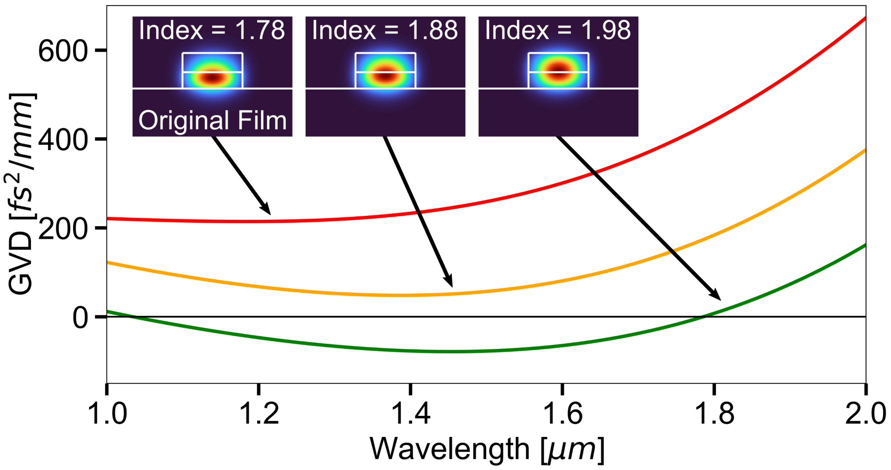

Fig. S6. Demonstration of anomalous GVD with our waveguide design when using higher index PECVD SiN films. The simulated waveguides have a cross section of 1500 nm X 730 nm (340 nm bottom LPCVD SiN layer; 400 nm top PECVD SiN layer). The index shown in the diagram is for a wavelength of 1550 nm.

To further prove our designs flexibility and robustness, we experimentally demonstrate anomalous GVD combs with our waveguide design using a higher index PECVD SiN film. We fabricate a bilayer waveguide ring resonator consisting of a 340 nm LPCVD SiN bottom layer and a 400 nm top PECVD SiN. We follow the exact same fabrication process detailed in the Methods Section, only we alter the PECVD parameters to yield a SiN film with a refractive index close to 2 at a wavelength of 1550 nm. Using a waveguide width of 1.5 μm and a ring radius of 200 μm, we generate the low noise, anomalous GVD comb shown in Figure S7 using roughly 180 mW on on-chip power. Like in the normal comb case, we see a distinct drop in the RF frequency noise once the microresonator comb is locked via an on-chip heater.

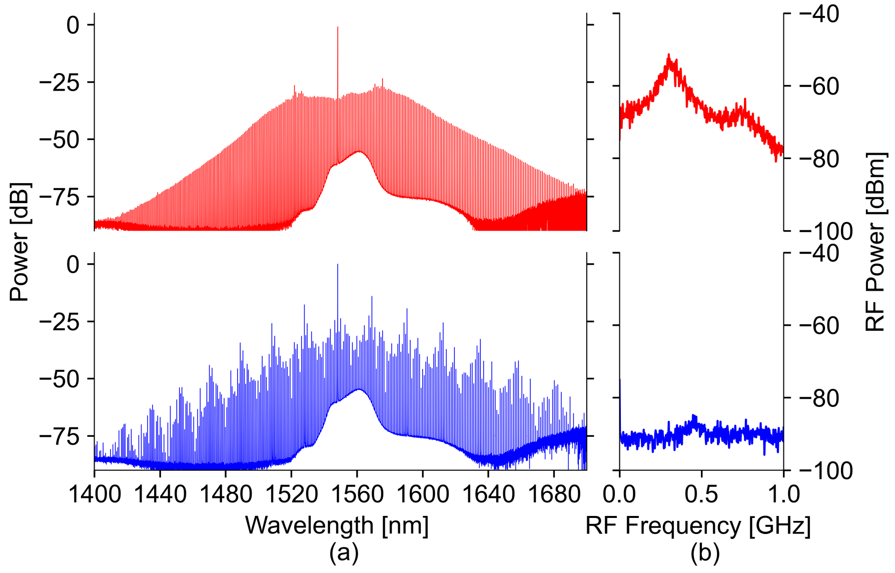

Fig. S7. (a) OSA spectrum of the unlocked (top) and locked (bottom) comb states of our bilayer waveguide resonator with a higher index PECVD SiN top film. (b) Associated RF frequency noise measured via an ESA.

5. LPCVD SIN MICRORESONATOR COMB AND COMPARISON TO BILAYER COMBS

We generate a comb with the fabricated LPCVD SiN waveguides to compare to our bilayer waveguide results. Figure S8 shows the OSA spectrum of a locked, anomalous comb generated in a 150 μm radius ring with a waveguide dimension of 1500 nm X 730 nm. The comb is generated using roughly 200 mW of on-chip power, again using the on-chip heater to lock the comb.

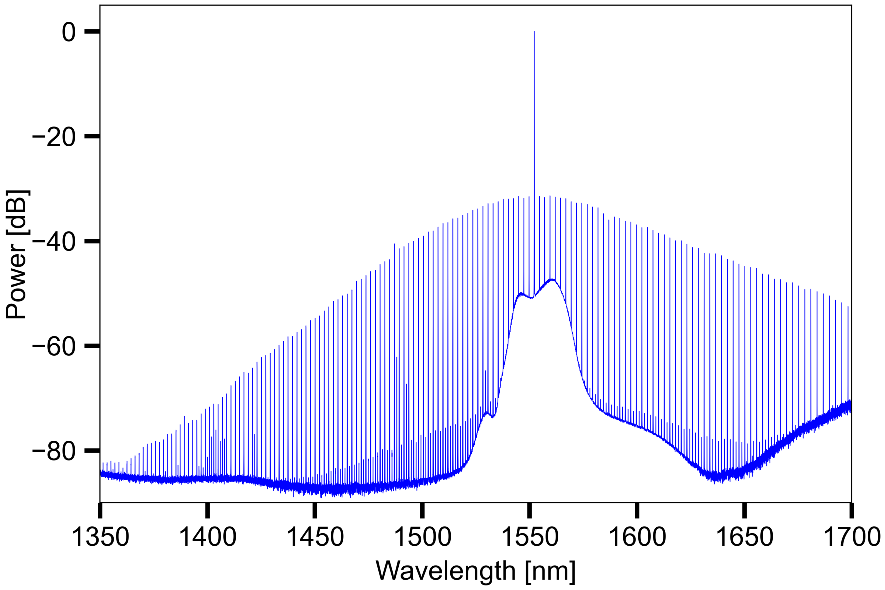

Fig. S8. OSA spectrum of a locked comb generated with an LPCVD SiN ring.

The LPCVD SiN comb yields the largest bandwidth. This can be accounted for in the bilayer scheme simply by adjusting the height of the higher index PECVD SiN slightly. Furthermore, the on-chip power required to generate both anomalous combs demonstrated are comparable. The efficiency of the combs, meanwhile, is very dependent on the resonator design, coupling regime, and loss [6]. As such, with appropriate design of the coupling and ring geometry, our bilayer design could be optimized for comb efficiency in the anomalous regime.

Though the low index SiN film does not allow anomalous GVD tuning, the normal GVD comb generated shows a relatively large bandwidth and a reasonable on chip pump power compared to other normal GVD comb demonstrations [7, 8]. The use of a top film with such a differing refractive index highlights the robustness in refractive index variation our bilayer approach can handle and still be used for nonlinear applications.

REFERENCES

1. K. Luke, A. Dutt, C. B. Poitras, and M. Lipson, "Overcoming Si_3N_4 film stress limitations for high quality factor ring resonators," *Optics Express*, vol. 21, no. 19, pp. 22 829–22 833, Sep. 2013, publisher: Optica Publishing Group. [Online]. Available: https://opg.optica.org/oe/abstract.cfm?uri=oe-21-19-22829
2. C. Joshi, J. K. Jang, K. Luke, X. Ji, S. A. Miller, A. Klenner, Y. Okawachi, M. Lipson, and A. L. Gaeta, "Thermally controlled comb generation and soliton modelocking in microresonators," *Optics Letters*, vol. 41, no. 11, pp. 2565–2568, Jun. 2016, publisher: Optica Publishing Group. [Online]. Available: https://opg.optica.org/ol/abstract.cfm?uri=ol-41-11-2565
3. K. Luke, Y. Okawachi, M. R. E. Lamont, A. L. Gaeta, and M. Lipson, "Broadband mid-infrared frequency comb generation in a Si_3N_4 microresonator," *Optics Letters*, vol. 40, no. 21, pp. 4823–4826, Nov. 2015, publisher: Optica Publishing Group. [Online]. Available: https://opg.optica.org/ol/abstract.cfm?uri=ol-40-21-4823
4. M. Yasaka *et al.*, "X-ray thin-film measurement techniques," *The Rigaku Journal*, vol. 26, no. 2, pp. 1–9, 2010.
5. I. Guler, "Optical and structural characterization of silicon nitride thin films deposited by PECVD," *Materials Science and Engineering: B*, vol. 246, pp. 21–26, Jul. 2019. [Online]. Available: https://www.sciencedirect.com/science/article/pii/S0921510719301497
6. J. K. Jang, Y. Okawachi, Y. Zhao, X. Ji, C. Joshi, M. Lipson, and A. L. Gaeta, "Conversion efficiency of soliton Kerr combs," *Optics Letters*, vol. 46, no. 15, pp. 3657–3660, Aug. 2021, publisher: Optica Publishing Group. [Online]. Available: https://opg.optica.org/ol/abstract.cfm?uri=ol-46-15-3657
7. B. Y. Kim, Y. Okawachi, J. K. Jang, M. Yu, X. Ji, Y. Zhao, C. Joshi, M. Lipson, and A. L. Gaeta, "Turn-key, high-efficiency Kerr comb source," *Optics Letters*, vol. 44, no. 18, pp. 4475–4478, Sep. 2019, publisher: Optica Publishing Group. [Online]. Available: https://opg.optica.org/ol/abstract.cfm?uri=ol-44-18-4475
8. Y. Zhang, S. Zhang, T. Bi, and P. Del'Haye, "Geometry optimization for dark soliton combs in thin multimode silicon nitride microresonators," *Optics Express*, vol. 31, no. 25, pp. 41 420–41 427, Dec. 2023, publisher: Optica Publishing Group. [Online]. Available: https://opg.optica.org/oe/abstract.cfm?uri=oe-31-25-41420
